# Relationship between future time perspective and academic self-efficacy: the chain mediating roles of personal growth initiative and learning engagement

**DOI:** 10.1186/s40359-025-03254-2

**Published:** 2025-09-18

**Authors:** Xiao-Yan Zhu, Shi-Min Chen, Hang Lyu

**Affiliations:** 1 School of Foreign Language, Guangdong University of Petrochemical Technology, Maoming, China; 2 Department of Psychology, Huaiyin Normal University, Huai’an, China; 3 Department of Psychology, Yangzhou University, Yangzhou, China

**Keywords:** Future time perspective, Academic self-efficacy, Personal growth initiative, Learning engagement, Chain mediating model

## Abstract

**Background:**

Academic self-efficacy is a key predictor of academic performance. As college students mature, they increasingly focus on their future prospects. This study intended to further examine the relationship between college students’ future time perspective (FTP) and academic self-efficacy (ASE) by introducing personal growth initiative (PGI) and learning engagement (LE) as two mediators from the perspective of positive psychology.

**Methods:**

A total of 1387 college students were surveyed using the General Future Time Perspective Scale for College Students, Personal Growth Initiative Scale-II, Learning Engagement Scale, and Learning Ability Self-Efficacy Subscale. The mediation analysis was performed using bias-corrected nonparametric percentile bootstrap test.

**Results:**

Personal growth initiative and learning engagement significantly mediated the effect of future time perspective on academic self-efficacy, with a chain mediating effect size of 53.2% and a total mediating effect size of 67.5%.

**Conclusion:**

Personal growth initiative and learning engagement serve as two important mediators in the relationship between future time perspective and academic self-efficacy among college students. It is recommended to implement career planning interventions for college students to strengthen their future time perspective, promote personal growth initiative, enhance learning engagement, and ultimately boost academic self-efficacy.

**Supplementary Information:**

The online version contains supplementary material available at 10.1186/s40359-025-03254-2.

## Introduction

The college stage is a critical period for acquiring specialized knowledge and skills. By gaining foundational expertise in their field, college students lay a solid foundation for their future career development. Academic self-efficacy (ASE) is defined as learners’ judgments about their abilities to successfully attain educational goals and is manifested as a concrete expression of self-efficacy in the academic context [1, 2]. A wealth of literature has highlighted the importance of academic self-efficacy for learning and subsequent academic performance. A meta-analysis of 53 surveys involving 28,894 Chinese students has showed that the correlation coefficient between academic self-efficacy and academic performance is 0.405 (*p* < 0.001) for urban students, and 0.554 (*p* < 0.001) for rural students [3]. Therefore, it is of significance to explore college students’ academic self-efficacy and its influencing factors.

The core task of college students is to explore various possibilities regarding romantic relationships, careers, and worldviews in the stage of emerging adulthood [4, 5]. They increasingly focus on the future, filled with both aspiration and confusion [6, 7]. The term “future time perspective (FTP)” is coined to describe an individual’s focus and anticipation regarding the future. It refers to an individual’s imagery, expectations, ideas, and beliefs about the future, as well as their perception of future needs and the relationship among the past, present, and future [8]. Future time perspective significantly influences individual behavior, serving as a guiding and motivating function. Future time perspective helps adolescents and emerging adults establish goals, formulate plans, and take action based on their expectations and evaluations of the future [9]. This process enables them to more effectively adapt to and transform their social environments. Simultaneously, individuals gain a deeper sense of meaning in life through this journey [10].

Previous studies have indicated that future time perspective significantly affects academic self-efficacy [11, 12]. A high level of future time perspective can help individuals manage their time more effectively [13], enhance learning engagement [14], maintain sustained passion and perseverance towards long-term goals, and bravely persist in overcoming difficulties and challenges encountered in learning [15], thereby improving individuals’ academic self-efficacy [11] and academic performance. This study aimed to further explore the relationship between future time perspective and college students’ academic self-efficacy by introducing personal growth initiative and learning engagement as two mediators, framed within the perspective of positive psychology and utilizing a chain mediation model.

Personal growth initiative (PGI) refers to the awareness of individuals regarding changes in their lives, prompting them to actively change and enhance themselves to adapt to these changes [16–18]. Unlike other concepts of personal growth such as post-traumatic growth, personal growth initiative emphasizes the proactive transformation and enhancement of the self. Personal growth initiative consists of two main components: cognitive and behavioral [17, 19]. The cognitive component refers to an individual’s understanding of how to change themselves and their sense of efficacy regarding self-change; the behavioral component refers to the active steps taken by individuals to enact self-change. Individuals with high levels of personal growth initiative actively and proactively change and enhance themselves, enabling them to better adapt to their environments and serving as simple predictors of subjective well-being, psychological well-being, and social well-being [19].

Individuals with high-level future time perspective have a clear image of their future, a good understanding of future needs, and a clear comprehension of the relationship between the past, present, and future [8]. They also possess a clear understanding of their possible selves [20]. They recognize the gap between their actual self and their possible self, which prompts them to be more proactive in changing and enhancing themselves to transform their possible self into their actual self. In other words, individuals with high-level future time perspective exhibit high-level personal growth initiative. Therefore, Hypothesis 1 is proposed.

H1: Future time perspective significantly predicts personal growth initiative.

Learning engagement (LE) refers to a sustained and positive state that students exhibit in their academic activities within a school context [21]. Learning engagement is a crucial indicator of students’ active learning status. To achieve personal changes and self-improvement, college students need to work hard in their studies. In other words, students with high personal growth initiative are likely to invest more in their learning in order to achieve personal change and enhancement [22]. Therefore, Hypothesis 2 is presented.

H2: Personal growth initiative significantly predicts learning engagement.

The cultivation of various abilities requires a significant investment of time and energy. The more effort students put into their studies, the better their reading skills [23], translation skills [24], problem-solving abilities [25], and innovative capabilities [26] become, which in turn enhances their academic self-efficacy. Therefore, Hypothesis 3 is proposed.

H3: Learning engagement significantly predicts academic self-efficacy.

**Fig. 1:**
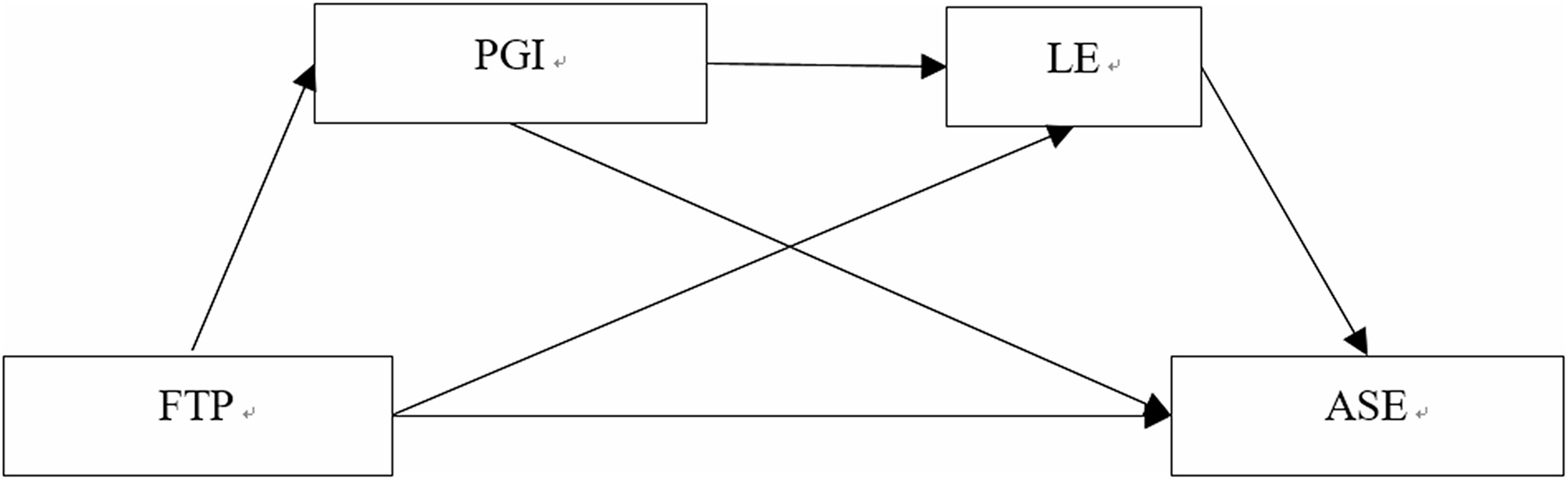
Hypothetical chain mediation model between FTP and ASE. Notes: FTP = future time perspective; PGI = personal growth initiative; LE = learning engagement; ASE = academic self-efficacy

Based on the analysis above, it is clear that future time perspective not only significantly predicts academic self-efficacy but also influences it through the indirect pathways of personal growth initiative and learning engagement. Therefore, Hypothesis 4 is presented. Accordingly, a hypothetical chain mediation model (Model 1) between future time perspective and academic self-efficacy is proposed (Fig. 1). This model includes three mediating effects: (1) the simple mediating effect of personal growth initiative between future time perspective and academic self-efficacy; (2) the simple mediating effect of learning engagement between future time perspective and academic self-efficacy; (3) the chain mediating effect of personal growth initiative and learning engagement between future time perspective and academic self-efficacy.

H4: Personal growth initiative and learning engagement play a chain mediating role between future time perspective and academic self-efficacy.

## Method

### Procedure

The following steps were taken in the survey. First, we obtained ethical approval from the Academic Ethics Committee at the first author’ affiliated institution. Second, we uploaded the test questionnaires to “Questionnaire Star”, a widely used survey platform in China, then embedded the QR code in the PowerPoint (PPT). Third, we recruited teachers from seven universities in China to assist with our study, including two key universities, two first-tier universities, and three second-tier universities. To ensure standardized implementation, we provided remote training on the testing procedures via phone calls and WeChat—a widely used messaging app in China. Fourth, the formal survey was conducted in class. Students were told that their participation in the study was anonymous and voluntary; that they were free to withdraw at any time and that their responses would be kept confidential. They were asked to provide written informed consent after the procedures had been fully explained. They then scanned the test QR code using WeChat and began to answer the questionnaire. After completing the questionnaire, students were given a little gift.

### Participants

Two lie-detection items were included in the test questionnaire: “I often grasp the lessons taught by the teachers in class well” and “I frequently have difficulty understanding the lessons presented by the teachers in class”. The Participant data were deemed invalid and excluded in cases of inconsistent responses to lie-detection items. A total of 1387 valid questionnaires were obtained (see supplementary materials Table S). The participants consisted of 722 boys and 665 girls. Their ages ranged from 17 to 28 years with a mean of 19.5 years (SD = 1.3). The sample contained 460 freshmen, 352 sophomores, 345 juniors, and 230 seniors. A total of 725 students majored in science and engineering, while 662 students pursued degrees in arts and humanities.

### Measures

#### Future time perspective

Future time perspective was assessed with 20-item General Future Time Perspective Scale for College Students [9]. The scale includes five dimensions: future imagery (e.g., “I have a relatively clear vision for the future”), long-term goal orientation (e.g., “I often remind myself not to forget the most important goals for the future”), future purpose (e.g., “I have set clear goals for my future”), future efficacy (e.g., “I believe my future is bright”), and behavioral commitment (e.g., “I achieve my goals by gradually progressing and completing my plans on time”). Responses were made on a 5-point Likert scale ranging from 1 (*strongly disagree*) to 5 (*strongly agree*) in the last year. A higher score indicates a greater level of future time perspective. The reliability of the scale in this study was Cronbach’s α = 0.913. The result of confirmatory factor analysis (CFA) indicates the scale has acceptable structure validity: χ^2^/df = 4.15, CFI = 0.925, TLI = 0.917, RMSEA = 0.054.

#### Personal growth initiative

Personal growth initiative was measured with 16-item Personal Growth Initiative Scale-II [17], whose Chinese version was revised by Xu et al. (2019) [27]. This scale consists of four dimensions: readiness for change (e.g. “I figure out what I need to change about myself”), planfulness (e.g., “I set realistic goals for what I want to change about myself”), using resources (e.g., “I actively seek out help when I try to change myself.”), and intentional behavior (e.g., “I actively work to improve myself”). Responses were made on a 5-point Likert scale ranging from 1 (*strongly disagree*) to 5 (*strongly agree*) in the last year. A higher score reflects a greater level of personal growth initiative. The reliability of the scale in this study was Cronbach’s α = 0.886. The result of CFA indicates the scale has acceptable structure validity: χ^2^/df = 3.83, CFI = 0.930, TLI = 0.923, RMSEA = 0.053.

#### Learning engagement

Learning engagement was assessed with 17-item Learning Engagement Scale [28], whose Chinese version was revised by Fang et al. (2008) [29]. This scale comprises three dimensions: vigor (e.g., ‘When I get up in the morning, I feel like going to class’), dedication (e.g., ‘I’m enthusiastic about my study’), and absorption (e.g., ‘When I’m studying, I forget everything around me’). Responses were made on a 5-point Likert scale ranging from 1 (*strongly disagree*) to 5 (*strongly agree*) in the last year. A higher score indicates a greater level of learning engagement. The reliability of the scale in this study was Cronbach’s α = 0.933. The result of CFA indicates the scale has acceptable structure validity: χ^2^/df = 3.51, CFI = 0.948, TLI = 0.942, RMSEA = 0.042.

#### Academic self-efficacy

Academic self-efficacy was assessed with 11-item Learning Ability Self-Efficacy Subscale [30]. Example items were “I believe I am able to achieve good results in my studies”, “I think I am capable of solving problems that arise in my learning”. Responses were made on a 5-point Likert scale ranging from 1 (*strongly disagree*) to 5 (*strongly agree*) in the last year. A higher score reflects a greater level of academic self-efficacy. The reliability of the scale in this study was Cronbach’s α = 0.887. The result of CFA indicates the scale has acceptable structure validity: χ^2^/df = 3.75, CFI = 0.934, TLI = 0.9287, RMSEA = 0.052.

### Statistical methods

The descriptive statistics were performed by the software of SPSS 26.0. Product of coefficients method was used to test the mediating effect [31], and bias-corrected nonparametric percentile Bootstrap method was employed to assess the confidence interval by drawing 2000 bootstrap samples with replacement from the dataset [32, 33]. Structural equation modelling was constructed with the software of Mplus 7.4. The mediation effect size is calculated according to the ratio of the indirect effect to the total effect, that is, ab/(ab + c’) [34].

## Results

### Descriptive statistics and bivariate correlations

As shown in Table 1, future time perspective, personal growth initiative, learning engagement, and academic self-efficacy of college students are all significantly positively correlated with each other.

**Table 1:** Descriptive statistics and bivariate correlations

Variables	1	2	3	4
1.FTP	1			
2.PGI	0.797**	1		
3.LE	0.704**	0.755**	1	
4.ASE	0.637**	0.654**	0.714**	1
M	3.36	3.22	3.14	3.24
SD	0.61	0.64	0.77	0.74

Notes: FTP = future time perspective, PGI = personal growth initiative, LE = learning engagement, ASE = academic self-efficacy, ** *p* < 0.01

### Mediating effect of FTP on ASE

**Fig. 2:**
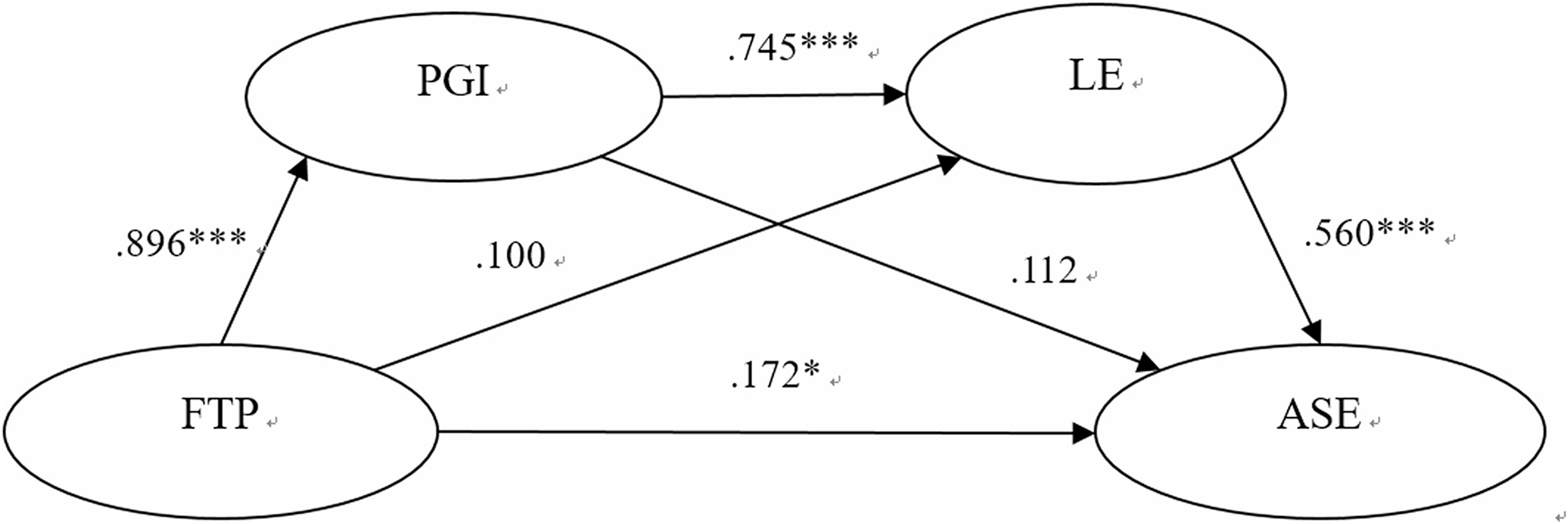
Chain mediation model between FTP and ASE

A structural equation model (SEM) was constructed according to hypothetical Model 1. The model fit indices all met the cut-off criteria (χ²/df = 4.79 ≤ 5.0, CFI = 0.911 ≥ 0.90, TLI = 0.903 ≥ 0.90, RMSEA = 0.065 ≤ 0.08), suggesting that the model was acceptable. As shown in Fig. 2, the standardized path coefficients all reached significant levels: future time perspective on personal growth initiative (*β* = 0.896, *p* < 0.001), personal growth initiative on learning engagement (*β* = 0.745, *p* < 0.001), and learning engagement on academic self-efficacy (*β* = 0.560, *p* < 0.001).

Table 2 indicates the confidence intervals and mediating effect sizes of future time perspective on academic self-efficacy. The simple mediating effect via the mediator of personal growth initiative ([-0.039, 0.235]) included 0, indicating that this mediating effect was not significant. The effect size for this simple mediation was 14.3%. Conversely, the chain mediating effect through the mediators of personal growth initiative and learning engagement ([0.285, 0.468]) did not contain 0, suggesting that the chain mediating effect was significant, with an effect size of 53.2%. In summary, the total mediating effect size through the mediator of personal growth initiative reached 67.5%.

**Table 2:** Confidence intervals and mediating effect sizes of FTP on ASE

Effect	Mediators	Effect values	95%CI	Effect sizes
Direct		0.172	[0.062,0.291]	24.5%
Indirect	PGI	0.100	[-0.039,0.235]	14.3%
	LE	0.056	[-0.015,0.128]	8.0%
	PGI-SE	0.374	[0.285,0.468]	53.2%
	Total	0.530	[0.420,0.634]	75.5%
Total		0.702		

## Discussion

### Relationship between FTP and ASE

The objective of this study was to further explain the relationship between future time perspective and academic self-efficacy by introducing personal growth initiative and learning engagement as two mediators among college students. Descriptive statistical analysis showed that the mean of future time perspective was greater than the median, and it had the highest mean among the four variables (Table 1). This finding was consistent with previous research [35, 36], indicating that college students possessed a high level of future time perspective. The chain mediation analysis revealed that future time perspective affected academic self-efficacy through one chain mediation pathway and two simple mediation pathways.

#### Chain mediation pathway

Future time perspective showed a strong correlation with personal growth initiative (Table 1), and the standardized path coefficient for future time perspective on personal growth initiative was notably high (Fig. 2), indicating that future time perspective had a very significant predictive effect on personal growth initiative, supporting H1. This result suggested that individuals with a high-level future time perspective tend to be more proactive in pursuing self-improvement and personal development.

The standardized path coefficient for personal growth initiative on learning engagement was great (Fig. 2), indicating that personal growth initiative had a very significant predictive effect on learning engagement, supporting H2. This result was consistent with previous research [22]. College students with a high level of personal growth initiative devote substantial time and energy to learning, actively overcoming various difficulties encountered in the learning process to achieve self-improvement.

The standardized path coefficient for learning engagement on academic self-efficacy was high (Fig. 2), indicating that personal growth initiative significantly predicted learning engagement, supporting H3. Previous research has focused on the impact of academic self-efficacy on learning engagement [37–40]. In contrast, this study examined the effect of learning engagement on academic self-efficacy. Through sustained engagement in learning processes and systematic development of multiple competencies, college students can cultivate strong academic self-efficacy. This finding carries significant implications for student development.

The 95% confidence interval for the effect of future time perspective on academic self-efficacy through personal growth initiative and learning engagement did not include 0 (Table 2), indicating that the chain mediation effect was significant, supporting H4. The chain mediating effect size was found to be 53.2%, indicating that this chain mediation pathway plays a significant mediating role between future time perspective and academic self-efficacy.

#### Simple mediation pathway via PGI

The correlation analysis revealed a strong association between PGI and ASE (Table 1). However, in the chain mediation model, the standardized pathway coefficient from PGI to ASE was not significant. This is because PGI does not directly affect ASE, but rather exerts its influence indirectly through learning engagement (Fig. 2). The non-significant pathway coefficient between PGI and ASE resulted in an insignificant simple mediation effect of PGI in the relationship between FTP and ASE. The simple mediation effect size via PGI was 14.3%, while the chain mediation effect size through both PGI and learning engagement reached 53.2%. This difference indicated that FTP had an impact on ASE mainly through the chain mediation pathway, whereas the simple mediating effect via PGI was relatively limited.

#### Simple mediation pathway via LE

Correlation analysis indicated a strong link between FTP and learning engagement (Table 1). However, in the chain mediation model, the standardized pathway coefficient of FTP on learning engagement was not significant. This is because FTP does not directly influence learning engagement; instead, it indirectly affects learning engagement through PGI (Fig. 2). The non-significant standardized pathway coefficient for FTP on learning engagement resulted in an insignificant simple mediation effect of learning engagement in the relationship between FTP and ASE. The simple mediation effect size via learning engagement was 8.0%, whereas the chain mediation effect size through both PGI and learning engagement reached 53.2%. This difference suggested that FTP primarily influenced ASE through the chain mediation pathway, whereas the simple mediating effect via learning engagement was relatively limited.

In sum, the total mediating effect size reached an impressive 67.5%. This highlighted that personal growth initiative and learning engagement played a crucial role in mediating the relationship between future time perspective and academic self-efficacy. In addition, the mediating effect sizes were greater than those observed with other mediators: time management disposition (38.14%) [13], perseverance (15.1%) [15], and learning engagement (57.71%) [14]. This further highlights the significance of the two mediators examined in this study.

### Highlights

This study yielded the following highlights. First, this study explored the mediating roles of personal growth and learning engagement as two mediators in the relationship between future time perspective and academic self-efficacy. Utilizing a chain mediation model from a positive psychology perspective, the research further investigated how future time perspective influenced academic self-efficacy.

Second, in contrast to previous research that has focused on the impact of academic self-efficacy on learning engagement, this study explored the reverse relationship—specifically, the effect of learning engagement on academic self-efficacy. This shift in focus carries significant implications for the growth and development of college students.

### Practical implications

This study has important practical significance in promoting college students’ academic efficacy by enhancing their future time perspective. College students can be supported in their career planning, especially by leveraging current AI technologies like machine learning to enhance their future time perspective [41–45]. First, college students can gain insights into future social development trends by discussions with teachers and distinguished professionals across various fields, attending lectures, and participating in part-time jobs. Second, an assessment of college students can be conducted, which includes not only their career interests, key strengths, and underlying values [46] but also their academic performance in different subjects, growth experiences, and family environment [41]. Third, machine learning, such as deep neural networks, can be used to assist in career selection and planning. Machine learning can simultaneously incorporate numerous predictive variables and capture complex patterns and associations in the data more effectively through nonlinear mapping and automatic feature learning [47, 48]. By employing machine learning, more suitable career options can be identified, and personalized career plans can be developed.

Through systematic career assessment and strategic planning, college students develop a more comprehensive understanding of their professional aspirations, personal needs, and the interconnectedness of their past experiences, present circumstances, and future goals. This enhances their future time perspective, which in turn encourages them to actively strive for self-improvement and increase their learning engagement, thereby boosting their academic self-efficacy.

### Limitations and future directions

Despite its contributions, some limitations of this study should be acknowledged. First, this study employs a self-report method, which has limitations such as potential inaccuracies in participants’ responses, lack of seriousness in answering, social desirability bias, and a tendency toward central tendency effects. Future research could consider using alternative methods to more objectively assess college students’ future time perspective and academic self-efficacy.

Second, the chain mediation model constructed in this study was validated using cross-sectional survey data, which essentially reflects a correlational study and cannot be used for strict causal interpretation. Future studies could conduct longitudinal surveys to build a longitudinal mediation model [49], thereby providing a better causal explanation for the relationship between future time perspective and college students’ academic self-efficacy.

Third, although the suggested measures to enhance college students’ future time perspective demonstrate theoretical potential, their effectiveness has yet to be verified. Future implementation and evaluation in practice are essential.

Fourth, the integrated application of AI has become an important trend in the development of various fields today. Currently, research and applications that utilize AI technology to optimize career assessment systems and assist in career selection and planning are still in the early stages. Future research could adopt a multidisciplinary approach to collaboratively address this critical issue.

Fifth, this study employs a chain mediation model to primarily explore the impact of future time perspective on academic self-efficacy, while failing to comprehensively consider other factors on academic self-efficacy. Various factors, including socioeconomic status and prior academic performance, significantly influence academic self-efficacy. Machine learning can simultaneously incorporate numerous predictive variables and better capture the complex patterns and relationships within the data through nonlinear mapping and automatic feature learning [47, 50]. Future research could utilize machine learning to further examine the combined effects of various factors on academic self-efficacy.

## Conclusion

Personal growth initiative and learning engagement significantly mediate the effect of college students’ future time perspective on academic self-efficacy, with a large chain mediating effect size and a total mediating effect size. Career assessments, particularly those enhanced by AI, can be offered to college students to support their career planning. This approach enhances their future time perspective, fosters their personal growth initiative, and increases their learning engagement, ultimately boosting their academic self-efficacy.

Table S Data for the relationship between future time perspective and academic self-efficacy.

## Supplementary Information

Below is the link to the electronic supplementary material.

Supplementary Material 1

## Data Availability

Data are provided in the supplementary file.
